# Modeling C3 glomerulopathies: C3 convertase regulation on an extracellular matrix surface

**DOI:** 10.3389/fimmu.2022.1073802

**Published:** 2023-01-18

**Authors:** Sofiya Pisarenka, Nicole C. Meyer, Xue Xiao, Renee Goodfellow, Carla M. Nester, Yuzhou Zhang, Richard J. H. Smith

**Affiliations:** ^1^ Molecular Otolaryngology and Renal Research Laboratories, Caver College of Medicine, University of Iowa, Iowa City, IA, United States; ^2^ Molecular Medicine Graduate Program, Caver College of Medicine, University of Iowa, Iowa City, IA, United States

**Keywords:** C3 glomerulopathies, extracellar matrix, complement regulation, C3 nephritic factor (C3Nef), factor H (FH), factor B (FB), C3 convertase

## Abstract

**Introduction:**

C3 glomerulopathies (C3G) are ultra-rare complement-mediated diseases that lead to end-stage renal disease (ESRD) within 10 years of diagnosis in ~50% of patients. Overactivation of the alternative pathway (AP) of complement in the fluid phase and on the surface of the glomerular endothelial glycomatrix is the underlying cause of C3G. Although there are animal models for C3G that focus on genetic drivers of disease, in vivo studies of the impact of acquired drivers are not yet possible.

**Methods:**

Here we present an in vitro model of AP activation and regulation on a glycomatrix surface. We use an extracellular matrix substitute (MaxGel) as a base upon which we reconstitute AP C3 convertase. We validated this method using properdin and Factor H (FH) and then assessed the effects of genetic and acquired drivers of C3G on C3 convertase.

**Results:**

We show that C3 convertase readily forms on MaxGel and that this formation was positively regulated by properdin and negatively regulated by FH. Additionally, Factor B (FB) and FH mutants impaired complement regulation when compared to wild type counterparts. We also show the effects of C3 nephritic factors (C3Nefs) on convertase stability over time and provide evidence for a novel mechanism of C3Nef-mediated C3G pathogenesis.

**Discussion:**

We conclude that this ECM-based model of C3G offers a replicable method by which to evaluate the variable activity of the complement system in C3G, thereby offering an improved understanding of the different factors driving this disease process.

## Introduction

C3 Glomerulopathies (C3G) are a group of ultra-rare complement-mediated renal diseases defined by specific histopathological findings on renal biopsy. The C3G definition includes presence of glomerulonephritis with C3-dominant immunofluorescence staining: C3 intensity must be at least two orders of magnitude more than any other immunoreactant. Electron microscopy (EM) is used to distinguish between the two major subtypes of C3G: Dense Deposit Disease (DDD) and C3 Glomerulonephritis (C3GN). DDD presents with extremely electron-dense, “sausage-shaped” deposits in the lamina densa of the glomerular basement membrane (GBM), a kidney-specific type of an extracellular matrix (ECM). In comparison, C3GN presents with subendothelial, subepithelial and/or mesangial deposits that are less electron-dense and have a less compact, “cloudy” appearance (1–3). The most important outcome associated with C3G diagnosis is the progression to end-stage renal disease (ESRD): ~50% of patients reach ESRD within 10 years of diagnosis (2, 4, 5). If kidney transplantation is offered, C3G recurrence in allografts is common (~60-80%), contributing to graft loss in ~50% of the cases (4, 6–10).

**Figure 1:**
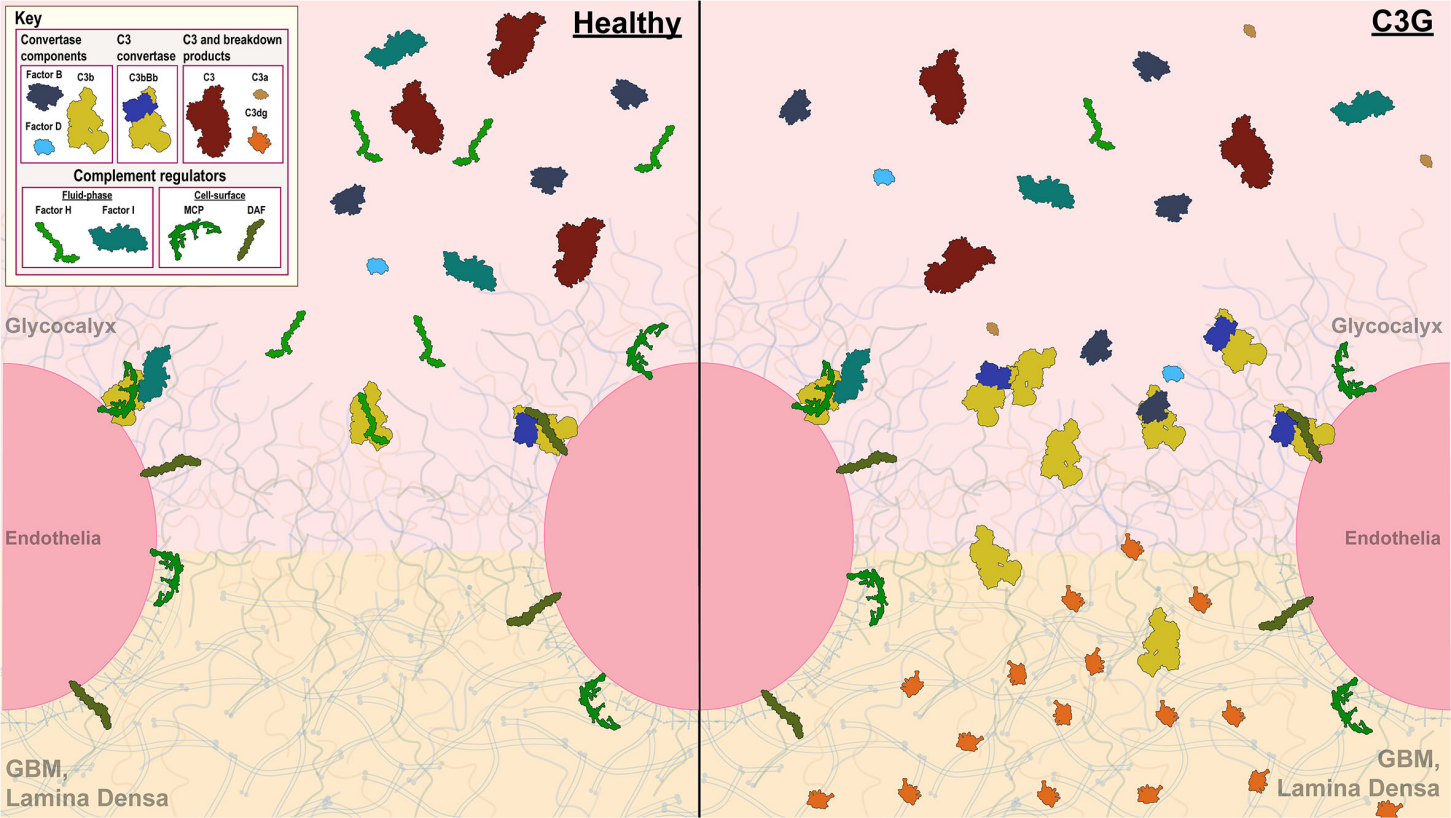
Complement control in the glomerular microenvironment. The glomerulus is the filtration unit of the kidney. Its filtration barrier is composed of the glycocalyx, glomerular endothelial cells (GEnC), glomerular basement membrane (GBM) and podocytes. The glycocalyx, a network of proteoglycans and glycoproteins, overlays highly fenestrated GEnCs. GEnC pores comprise ~20-50% of total cell surface area (11–16), allowing filtration of waste products through the glycomatrix (glycocalyx and the GBM), underlying podocytes and further into the Bowman’s capsule to be excreted as urine. Complement activity on GEnCs is controlled by both cell-bound (MCP and DAF depicted) and fluid-phase complement regulators (Factor H (FH) and Factor I (FI) depicted), while complement control over the GEnC pores relies on fluid-phase regulators alone. A healthy glomerular microenvironment exhibits adequate cell-surface and fluid-phase complement control thereby preventing injury to the GEnCs and complement deposition in the glycomatrix. In contrast, in the C3G glomerular microenvironment, fluid-phase complement dysregulation occurs (an example of C3G driven by FH deficiency depicted) in presence of adequate cell-surface complement control. Here, GEnCs are still protected by the cell-bound regulators, but the lack of fluid-phase complement control over the GEnC pores allows complement amplification and complement deposition to occur as reflected by changes in the lamina densa of the GBM.

C3G pathogenesis is primarily driven by dysregulation of complement in the circulation and/or glomerular microenvironment (**Figure 1**). Complement is an integral part of the innate immune system, responsible for pathogen clearance and recruitment of immune cells to the site of complement activation. Of the three complement-initiating pathways (classical, lectin, alternative), the alternative pathway (AP) is the main contributor to C3G pathogenesis. The AP is continuously activated at a low rate in a process known as tick-over, resulting in cleavage of complement component 3 (C3) into an anaphylatoxin C3a and an opsonin C3b, which deposits on pathogen and self surfaces to drive formation of C3 convertase of the AP, C3bBb (17, 18). This process is tightly controlled by regulators of complement activation (RCA) which control complement activity to prevent injury to the host. In C3G, C3 convertase regulation is impaired, resulting in complement deposits in the renal glomeruli. While the source of disease is unknown in a subset of C3G cases (~35-40%), C3 convertase dysregulation and the subsequent development of disease is driven by acquired (~40-50%) or genetic drivers (~15-25%) in most (4, 19). Known drivers act on different parts of the complement cascade. For example, acquired drivers such as C3 nephritic factors (C3Nefs) stabilize C3 convertase (18, 20, 21), while genetic drivers like mutations in the *CFH* gene decrease inhibition of complement activity (22–24). Understanding the molecular processes underlying complement dysregulation is imperative for patient-specific management of C3G (2, 25, 26).

End organ renal damage begins in the glomeruli, which are high-flow, high-pressure capillary beds. The glomerular endothelial cells (GEnC) are highly fenestrated, with fenestrae comprising ~20-50% of total GEnC surface area (11–16). Complement control over the fenestrae depends on fluid-phase RCA proteins, which bind to heparin sulfate proteoglycans and sialic acids in the overlaying glycocalyx (27–30). Factor H (FH) and its related proteins (FHRs) are examples of the most important fluid-phase RCAs responsible for complement control in the glomeruli (22, 31, 32). Ultimately, it is the dysregulation of complement control in the fluid phase and at the glycomatrix (glycocalyx and the GBM) surface that gives rise to C3G.

We therefore developed an *in vitro* extra-cellular matrix (ECM) based model to test fluid phase complement regulation. Our goal was to model normal regulation of the C3 convertase on ECM surface and then determine how acquired and genetic drivers of C3G impact convertase activity. These studies advance our understanding of C3G pathogenesis, provide a diagnostic tool to monitor patient-specific complement dysregulation, and, potentially, may become a screening tool to test complement therapeutics based on a patient’s complement profile.

## Materials and methods

### Patient cohort selection

Six patients were selected from our C3G research cohort. All patients had biopsy-proven C3G and sufficient purified Immunoglobulin G (IgG) samples to complete all assays multiple times. Selection was based on complement biomarker data (**Table 1**). Three C3G patients had elevated C3Nef activity (>20% activity as determined by C3CSA, C3Nef+), and three C3G patients had normal C3Nef activity (≤20%, C3Nef-). Control normal human serum (NHS) IgG was purified from pooled sera of persons with no history of renal disease. All patients gave informed consent before donating samples and were enrolled in this study under the guidelines approved by the institutional review board of the University of Iowa.

**Table 1:** Patient biomarkers.

	Patient #	C3Nef (C3CSA) (<20%)	Factor H Autoantibodies FHAA (<200 AU)	Factor B Autoantibodies FBAA (<200 AU)
C3Nef + patients	P1	3+ (67%)	<50	<50
	P2	1+ (40%)	<50	<50
	P3	1+ (25%)	<50	77
C3Nef + patients	P4	Negative (17%)	<50	<50
	P5	Negative (15%)	<50	<50
	P6	Negative (12%)	<50	55

### IgG purification

Patient IgG was purified using the Melon Gel IgG Purification Kit (Thermo Scientific, Rockford, IL) according to the manufacturer’s instructions (33) and adjusted to 0.75 mg/ml.

### C3Nef activity assay

C3 Convertase Stabilizing Assay (C3CSA) was performed as described previously (18). Briefly, AP C3 convertase was formed on the surface of sheep erythrocytes (SE), followed by adding patient-purified IgG to C3 convertase coated SE. Next, C3 convertase was allowed to decay for 20 and 60 minutes. At each time point, 50 ul was removed and mixed with rat EDTA serum, which served as a source of terminal complement components. C3CSA activity was reported as a function of the degree of hemolysis at 20 minutes as measured by OD at λ415.

### Generation of recombinant FB and FH mutants

Recombinant FB and FH proteins were obtained through GeneArt, a division of ThermoFisher Scientific (Regensburg, Germany), as described previously (24). Briefly, the DNA coding region of select FB and FH variants with His-tag at the C- terminus was synthesized and cloned into a mammalian expression vector. Plasmids were then transfected into Expi 293 cells, followed by purification of the resulting Fb and FH protein directly from the culture supernatants using Ni2+ columns. After purification, all proteins were adjusted to 1 mg/mL in PBS and stored at -80°C until use (24).

### C3 convertase formation assay

**Figure 2:**
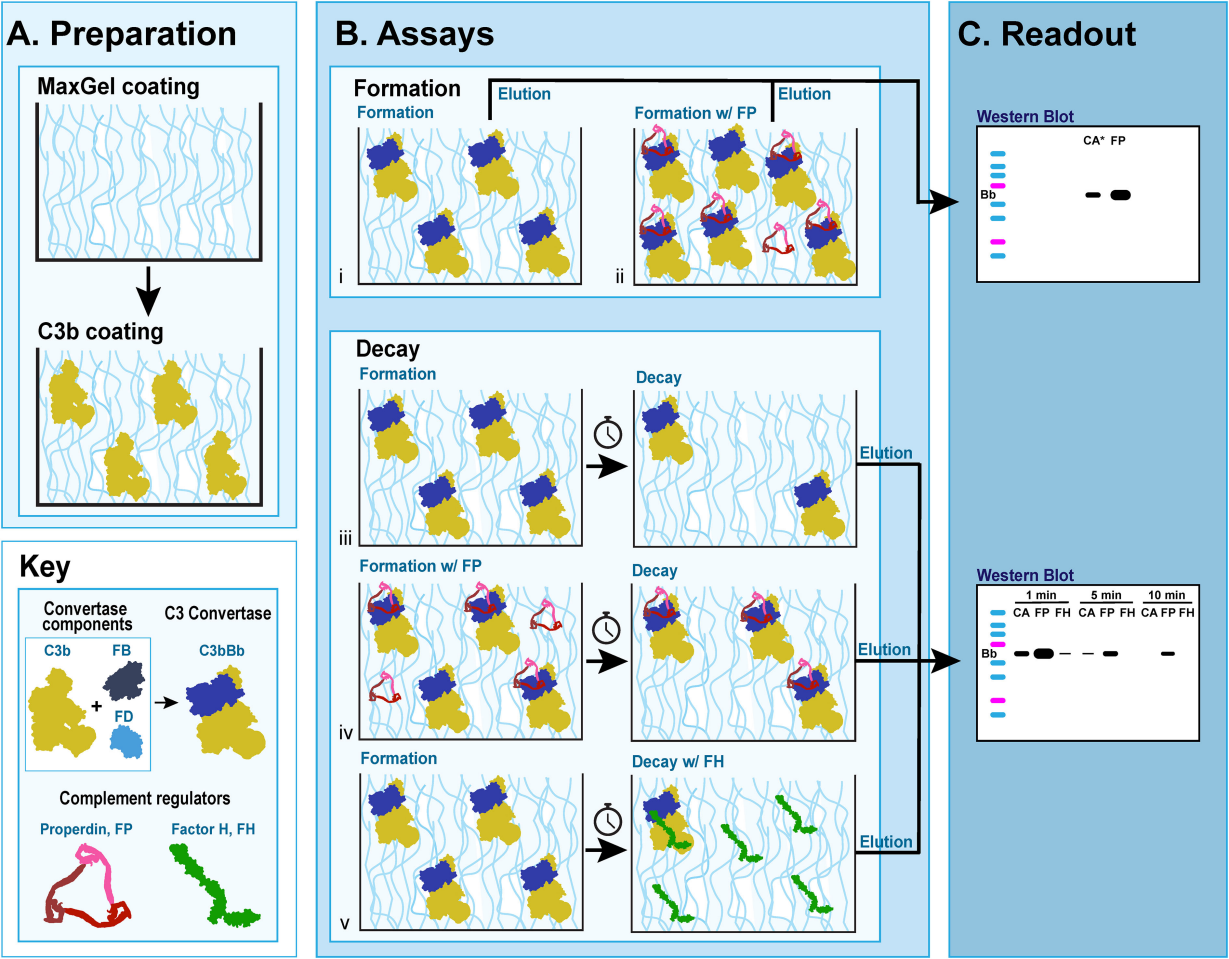
C3 convertase assays on MaxGel surface. **(A)** Preparation for all assays involves coating microtiter plates with MaxGel (ECM) followed by coating with C3b. **(B)** Formation assay: Factor B (FB) and Factor D (FD) are added to C3b-coated MaxGel, subsequently generating C3bBb over the course of 10 min in the absence (i) or presence (ii) of Properdin. Decay assay: C3bBb is generated as described above in absence (iii, v) or presence of Properdin (iv), and then allowed to decay over time naturally (iii, iv) or in presence of Factor H (v). **(C)** The amount of C3 convertase present on MaxGel surface at the time of elution is quantitated by western blot and observing the Bb complement fragment.

The C3 convertase formation assay (C3CFA) is a novel assay that measures the amount of C3 convertase formed on MaxGel™ (Sigma-Aldrich, St. Louis, MO) in the presence or absence of RCAs (**Figure 2B** i-ii). All complement proteins were obtained from Complement Technology Inc., Tyler, TX.

#### Preparation

96-well ELISA microtiter plates were coated with MaxGel (100 ul of 1:4 MaxGel:1xELISA coating buffer (Bio-Rad Laboratories, Inc., Hercules, CA)). Conditions were prepared in duplicate. Plates were incubated overnight (o/n) at 4°C. The following day, plates were washed with 1xPBS x3, then blocked with 4%BSA in 1xPBS for two hours at room temperature. Plates were then washed with 1xPBS x3 and wells were incubated with purified C3b (50 ul at 130 ug/ml) in 1xELISA coating buffer for at 4°C 2 o/n. C3b concentration was approximated using the normal physiological concentration of C3 (normal range, 900-1800 ug/ml) and was set at 1/10 of this value (concentration used, 1300 ug/ml); all other complement proteins and human IgG were used at 1/20 of their respective physiological concentrations (**Table 2**).

**Table 2:** Concentration of complement proteins in circulation.

Protein	Normal Range, ug/ml	Concentration used, ug/ml	Physiological concentration scaling factor	Assay concentration, ug/ml
C3	900-1800	1,300	1/10	130
Factor B	188-219	210	1/20	10.5
Factor D	1-26	2	1/20	0.1
Factor H	116-562	450	1/20	22.5
Properdin	17.5-25.2	20	1/20	1
Purified IgG	7,000-16,000	15,000	1/20	750

#### Assays

Fresh assay buffer (AB) was used for each experiment (8.1mM Na2HPO4, 1.8mM NaH2PO4, 0.05%Tween20, 75mM NaCl, 10mM MgCl_2_, 2% BSA in PBS, distilled H_2_O, pH = 7.0). Factor B (FB, 10.5 ug/ml) and Factor D (FD, 0.1 ug/ml) were added. 50 ul was added to each well (p/w). The same composition of reagents was used for all experiments.

C3b-coated wells were washed three times with AB to remove unbound C3b. C3 convertase was then formed by incubating the wells with control (FB+FD) or experimental (FB+FD+Reactant) assay mixtures (50 ul p/w) for 10 min at 37°C. Wells were washed with AB x3 to ensure that only MaxGel-bound C3 convertase remained.

#### Readout

Elution buffer (10mM EDTA and 1%SDS) was added to the plates at 25 ul p/w, followed by 1h incubation at room temperature on an orbital shaker. Eluants from duplicate wells were pooled, 35 ul of the pooled eluant was then added to 35 ul of 2x Laemmli Sample Buffer (Bio-Rad Laboratories, Inc., Hercules, CA) and heated at 95°C for 15 min in preparation for SDS-PAGE.

### C3 convertase decay assay

The C3 convertase decay assay (C3CDA) is a novel assay that measures the rate of decay of C3 convertase formed on MaxGel™ in presence or absence of RCAs or decay of C3 convertase alone (**Figure 2B** iii-v). Preparation and assay steps of the C3CFA protocol were followed for each timepoint assessed. Washed plates were incubated with AB alone or AB+Reactant (50 ul p/w) for a determined period at 37°C, then washed with AB x3. The readout for C3CDA was identical to the readout for C3CFA. The impact of properdin, FH and C3Nefs on C3 convertase decay was assessed.

### Visualization *via* SDS-PAGE and western blotting

Samples were separated by 10% SDS-PAGE gel (Bio-Rad Laboratories, Inc., Hercules, CA), and transferred to nitrocellulose membrane. Membranes were blocked with 5% skim milk in 1x PBST (1xPBS, 0.075% Tween20) at 4°C o/n, then incubated with a mouse monoclonal Factor B antibody specific for the Bb subunit (1:200 in 5% BSA, 1xPBST; Santa Cruz Biotechnology, Inc., Dallas, TX; F-7: sc-271636) at 4°C o/n. Membranes were washed x3 with 1xPBST, followed by a 2-hour incubation at room temperature with polyclonal HRP-conjugated goat anti-mouse secondary antibody (1:4000, Jackson ImmunoResearch Inc, West Grove, PA, 115-035-062, RRID: AB_2338504, in 5% BSA, 1xPBST). Membranes were washed x5 in 1xPBST, then incubated with SuperSignal West Pico PLUS Chemiluminescent Substrate (Thermo Scientific, Rockford, IL) for 1 min. Protein bands were visualized using Classic X-ray Film (Research Products International, Mt. Prospect, IL).

### Quantification and normalization of western blot data

Western blots were quantified using ImageJ (http://imagej.nih.gov/ij/; National Institutes of Health, Bethesda, MD). C3 convertase alone (CA) at the first assayed timepoint (as indicated in the legend) was set as 1; all other values in a replicate were normalized to this value.

### Statistical analyses

Statistical analyses were performed using GraphPad Prism 8 (GraphPad Software, San Diego, CA). All experiments were repeated a minimum of three times. Student’s t-test and one-way ANOVA with Dunnett’s multiple comparisons test were used to assess the difference between conditions. Half-life in decay assays was determined by fitting a curve with non-linear regression using second order polynomial equation. Difference was considered statistically significant at *P* ≤ 0.05.

## Results

### Validation of the method

#### C3 convertase assembles, decays on ECM surface and is regulated by RCA proteins

**Figure 3:**
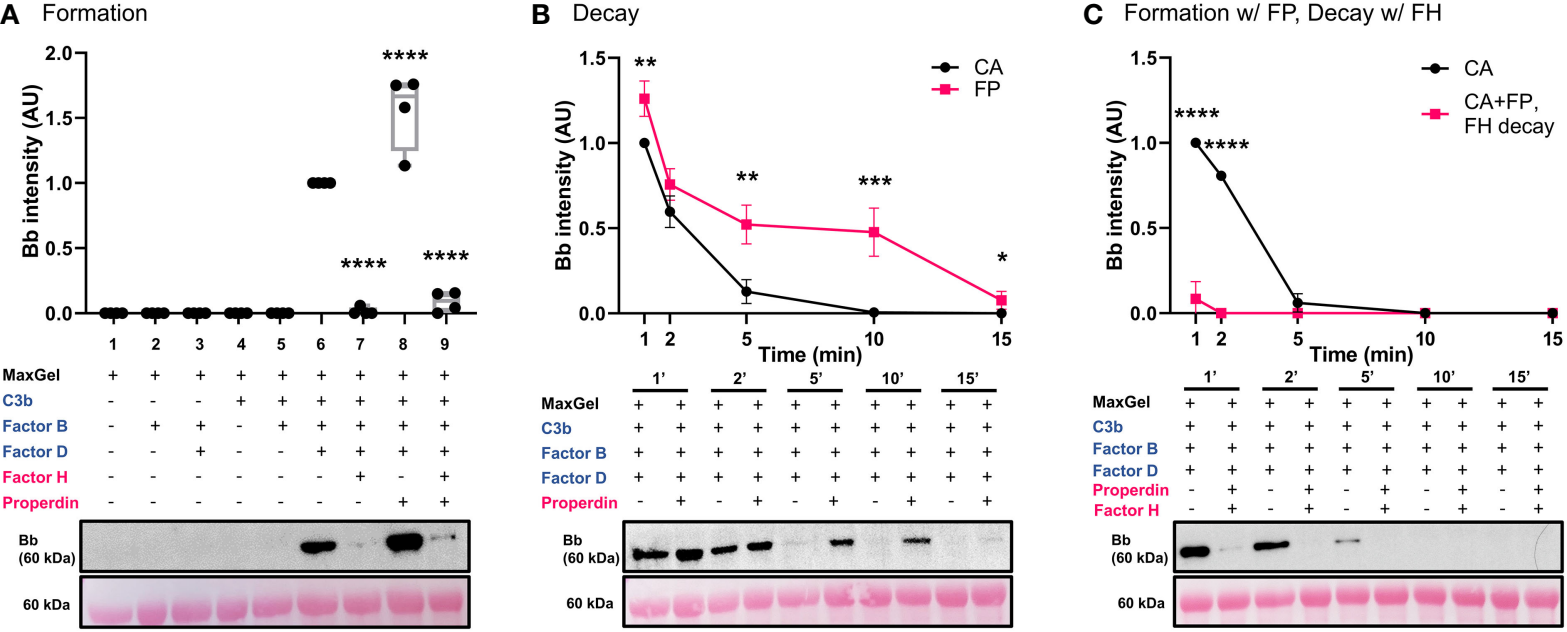
C3 convertase assembles, decays on ECM surface and is regulated by RCA proteins. **(A)** Western blot analysis of C3 convertase formation on ECM surface. MaxGel does not contain complement proteins necessary to form C3 convertase (lanes 1-5). C3 convertase alone (CA) forms in presence of C3b, FB and FD (lane 6), and is regulated by inhibitory FH (lane 7), stabilizing FP (lane 8) or a mix of FH+FP (lane 9). Results were normalized to CA and presented as relative protein expression. Box plots show median, 1^st^ and 3^rd^ quartile ranges, and the individual data points of four independent experiments. Significance calculated for CA. **(B)** Western blot analysis of timed decay (1-15 min) of CA formed alone or in presence of Properdin. FP increases C3bBb half-life more than 2-fold (CA *t_1/2 =_* 3.1min; FP *t_1/2 =_* 7.4min). **(C)** Western blot analysis of timed decay (1-15 min) of CA formed alone or in presence of properdin (C3bBb(P)), followed by FH-mediated decay of C3bBb(P). FH increases decay of C3bBb(P) (CA *t_1/2 =_* 3.2min; C3bBb(P)+FH *t_1/2_* <1min). Results were normalized to CA at 1 min and presented as relative protein expression. Ponceau S Staining was used as a measure of total protein load. Mean ± SD of four independent experiments. Significance calculated for CA at respective timepoints. * P ≤ 0.05, ** P ≤ 0.01, *** P ≤ 0.001, **** P ≤ 0.0001.

A novel *in vitro* model of complement activity and regulation was designed to assess C3 convertase activity on ECM surface (**Figure 2**). We first ensured that MaxGel, the ECM used as a base for all the assays, does not contain complement proteins capable of forming C3 convertase. To do so, we separately added necessary components of C3 convertase (C3b, FB, FD) or their combinations to MaxGel-coated wells (**Figure 3A**, lanes 1-5). Further, we demonstrated that C3bBb was only able to form on MaxGel surface in presence of all necessary components (**Figure 3A**, lane 6) and its formation was inhibited by Factor H (FH) and stabilized by Properdin (FP) (**Figure 3A**, lanes 7-8). Together, these experiments show that C3 convertase forms on MaxGel surface and that complement regulators act in the physiologically expected manner.

Properdin, the only known positive regulatory protein of the AP of complement, is capable of increasing the half-life of the C3 convertase up to 10 times (34). We hypothesized that physiological amounts of FP would stabilize C3bBb on MaxGel surface. To test this hypothesis, we assessed the differences in C3bBb decay rates when formed alone (CA) or in presence of FP (C3bBb(P)) (**Figure 3B**), following natural decay rates. Addition of FP led to 2.4-fold increase in convertase half-life (FP *t_1/2 =_* 7.4min) when compared to untreated control (CA *t_1/2 =_* 3.1min). Notably, though not statistically significant, some C3bBb(P) complexes were still present after 15 min of decay. C3bBb(P) decayed rapidly in the presence of FH (CA *t_1/2 =_* 3.2min), with complexes disappearing within 2 minutes (**Figure 3C**).

#### FH inhibits C3 convertase formation on ECM surface in dose-dependent manner

**Figure 4:**
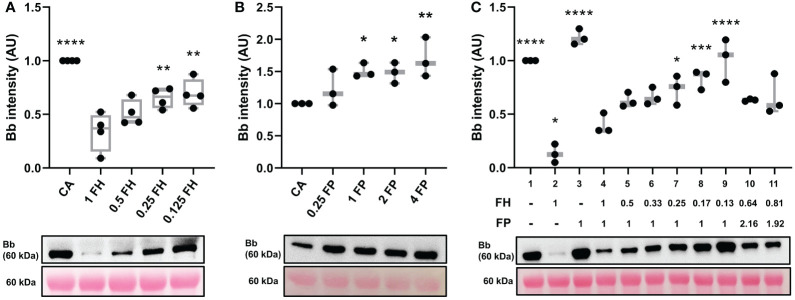
FH inhibits C3 convertase formation in dose dependent manner. **(A)** Western blot analysis of C3 convertase formed in presence of varying amounts of FH. Decreasing the concentration of FH (lanes 3-5) leads to decreased inhibition of C3 convertase formation in dose-depended manner, as compared to the normal physiological concentration of FH (lane 2). Box plots show median, 1^st^ and 3^rd^ quartile ranges, and the individual data points of four independent experiments. **(B)** Western blot analysis of C3 convertase formed in presence of varying amounts of properdin. Increasing the concentration of FP (lanes 3-5) leads to increased formation of C3 convertase in dose-depended manner, as compared to convertase alone (CA, lane 1). **(C)** Western blot analysis of C3 convertase formed in presence of decreasing FH : FP ratio. Decreasing the ratio of FH : FP (lanes 5-9) leads to decreased FH inhibition of complement formation in dose-dependent manner. Low FH : FP ratios allow for a significant increase (lanes 7-9) in convertase formation as compared to the physiological FH : FP ratio (lane 4). FH and FP biomarkers of two C3G patients with elevated FP levels from MORL C3G cohort are shown in lanes 10 (FH: 287ug/mL, FP: 43.2 ug/mL) and 11 (FH: 363 ug/mL, FP: 38.4 ug/mL). Results were normalized to CA and presented as relative protein expression. Ponceau S Staining was used as a measure of total protein load. Box plots show median, 1^st^ and 3^rd^ quartile ranges, and the individual data points of three independent experiments. Significance calculated for CA. * P ≤ 0.05, ** P ≤ 0.01, *** P ≤ 0.001, **** P ≤ 0.0001.

Factor H can disrupt formation of C3 convertase, perform decay acceleration activity (DAA) on already existing C3bBb complexes and act as a cofactor for another complement inhibitor, Factor I (FI) (32, 35, 36). Here we used the convertase formation assay to assess the impact of FH on C3 convertase formation. Decreasing concentrations of FH increased C3 convertase formation in dose-dependent manner (**Figure 4A**). Similarly, increasing concentrations of stabilizing properdin promoted dose-dependent increase in C3bBb formation (**Figure 4B**).

We modeled a range of Factor H:Properdin ratios by decreasing FH concentration while maintaining FP concentration to determine how changes in the relative ratio affect C3 convertase regulation on ECM surface (**Figure 4C**, lanes 4-9). A significant difference was observed at a ratio of FH : FP 0.25:1 (lane 7) which corresponds to 112.5 ug/ml FH and 20 ug/ml FP. We also modeled FH : FP ratios from two C3G patients with very high FP values (**Table 1**) (lanes 10-11). Neither reached significance when compared to the normal physiological ratio (lane 4, **Figure 4C**).

### Assessment of genetic drivers of C3G

#### Genetic variation in *CFB* may lead to variation in C3 convertase activity and regulation

**Figure 5:**
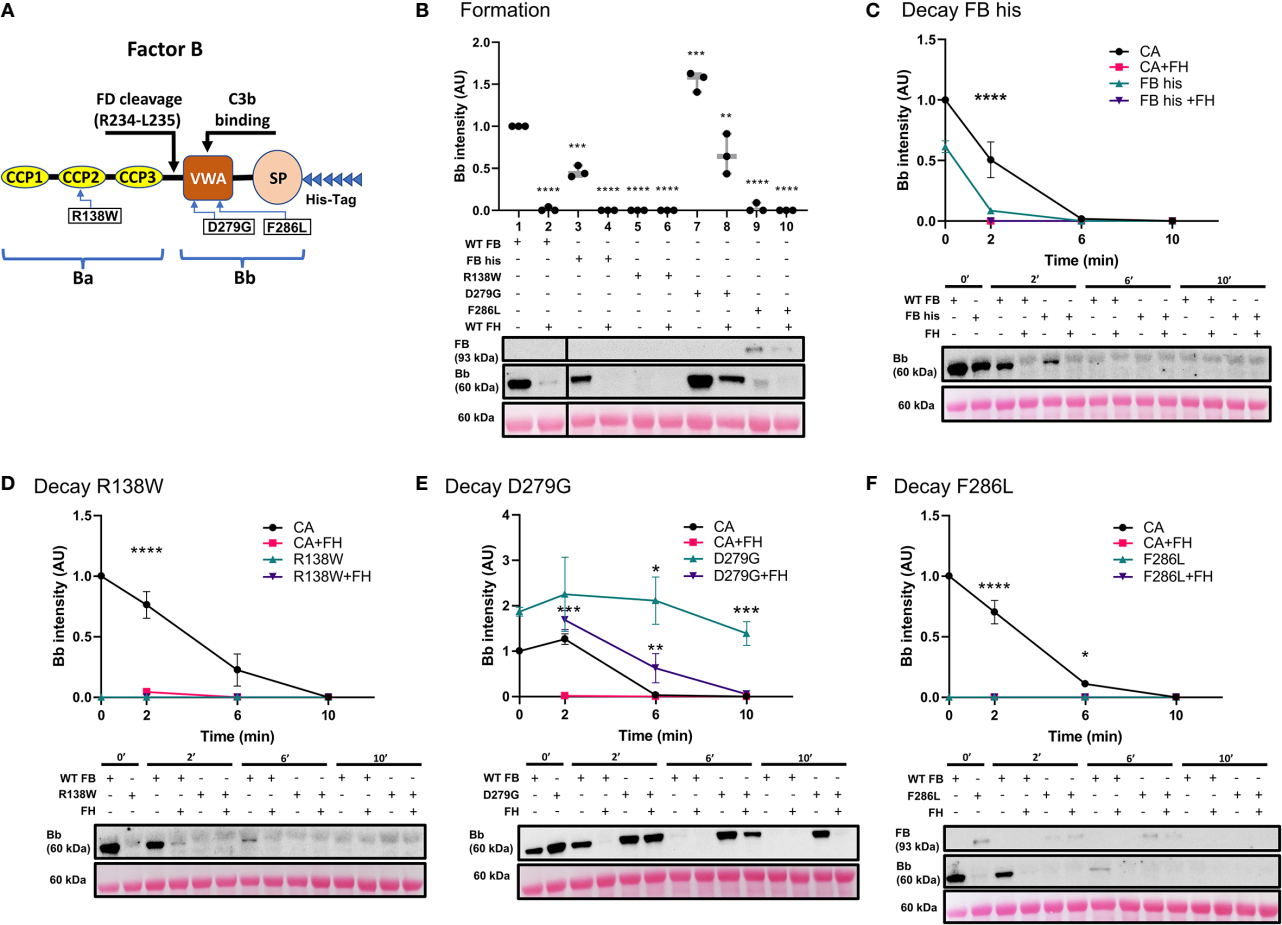
Variants in *CFB* can affect its affinity for C3b binding and rate of C3 convertase decay in presence/absence of FH. **(A)** Schematic of recombinant FB proteins, blue arrows denoting the positions of specific mutants. **(B)** Western blot analysis of C3 convertase formation. Recombinant WT FB (FB his) shows decreased ability to form C3 convertase (lanes 3) as compared to WT FB (CA, lanes 1). There is no formation in presence of FH (lanes 2 and 4). Both p.Arg138Trp (lanes 5-6) and p.Phe286Leu (lanes 9-10) fail to form C3bBb in absence/presence of FH. p.Asp279Gly increases C3 convertase formation ~1.5-fold (lane 7) as compared to CA (lane 1) and is able to form C3bBb even in presence of FH (lane 8). Results were normalized to CA and presented as relative protein expression. Box plots show median, 1^st^ and 3^rd^ quartile ranges, and the individual data points of three independent experiments. Significance calculated for WT FB. **(C)** Western blot analysis of CA formed using WT FB or FB his, then decayed in presence/absence of FH. Decrease in C3 convertase at 0 and 2 min is observed as compared to WT FB. **(D)** Western blot analysis of CA formed using WT or p.Arg138Trp, then decayed in absence/presence of FH. No mutant convertase activity is observed at 0 min as compared to WT FB. **(E)** Western blot analysis of CA formed using WT or p.Asp279Gly, then decayed in absence/presence of FH. Decrease in natural and FH-mediated decay of p.Asp279Gly C3bBb is observed as compared to WT FB at all timepoints. **(F)** Western blot analysis of CA formed using WT or p.Phe286Leu, then decayed in absence/presence of FH. No mutant convertase activity is observed at 0 min as compared to WT FB. For all decay experiments, results were normalized to CA at 0 min and presented as relative protein expression. Ponceau S Staining was used as a measure of total protein load. Statistical significance shown for comparisons between CA/recombinant protein and CA+FH/recombinant protein+FH respectively at the same timepoint. Mean ± SD of three independent experiments. * P ≤ 0.05, ** P ≤ 0.01, *** P ≤ 0.001, **** P ≤ 0.0001.

Three variants in *CFB* were assessed using our ECM-based model: one in the region encoding for the CCP2 subunit of Ba and two in the VWA subunit of Bb (**Figure 5A**). Convertase formation was significantly less with recombinant WT FB (FB his) (**Figure 5B**, lane 3) as compared to WT FB (lane 1); addition of FH prevented convertase formation entirely (lanes 2 and 4). The p.Arg138Trp failed to form active C3 convertase alone (lane 5) as well as in presence of FH (lane 6). The p.Asp279Gly, a gain-of-function mutation characterized by a high affinity for C3b and resistance to natural and FH-assisted decay (37–39), resulted in increased amounts of C3 convertase formation (lane 7). FH was not able to prevent C3 convertase formation by p.Asp279Gly (lane 8). No active C3bBb was observed for the p.Phe286Leu (lanes 9-10), but C3bB formed in absence and presence of FH, lanes 9-10).

The C3 convertase decay assay was used to assess the mutants’ natural and FH-mediated decay. As predicted from the results of the formation assay, FB his showed significantly less C3bBb after 2 min as compared to WT FB (FB his *t_1/2_* ~0.24 min, CA *t_1/2 =_* 2.2*_ min_*). Addition of FH resulted in complete decay of C3 convertase at 2 min (**Figure 5C**). Both p.Arg138Trp (**Figure 5D**) and p.Phe286Leu (**Figure 5F**) failed to form C3bBb, thus the observed absence of C3 convertase at 0 and 2 min of decay with or without FH was expected. C3bB resulting from the p.Phe286Leu was present after 10 min of natural and FH-mediated decay. Natural decay of C3bBb formed with p.Asp279Gly was significantly slower as compared to WT FB (D279G t_1/2_>10 min, CA t_1/2 =_ 4.2min). Decaying p.Asp279Gly C3 convertase with FH resulted in decreased DAA (D279G+FH t_1/2 =_ 6.6_ min_), with convertase complexes still seen at 6 min (**Figure 5E**).

#### Pathogenic variation in SCR 1-3 of *CFH* leads to decreased inhibitory function of FH on ECM surface

**Figure 6:**
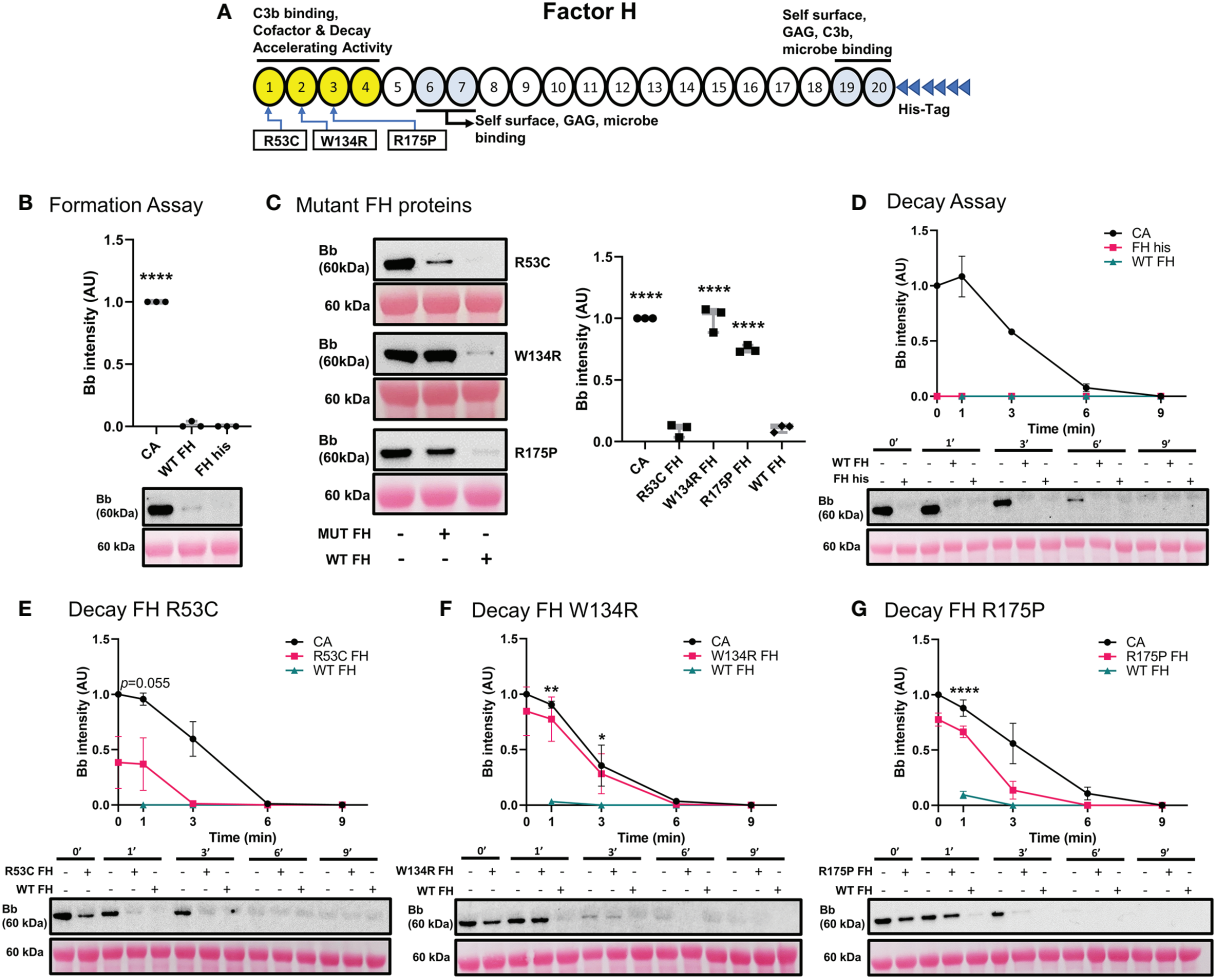
Variants in *CFH* can affect its ability to inhibit C3 convertase formation and promote Decay Acceleration Activity. **(A)** Schematic of recombinant FH proteins, blue arrows denoting the positions of specific mutants. **(B)** Western blot analysis of C3 convertase formed alone (CA), or in presence of WT FH or recombinant WT FH (FH his). Recombinant WT FH inhibits C3bBb formation to the same degree as WT FH. **(C)** Western blot analysis of CA formed alone, or in presence of recombinant FH variants or WT FH. p.Arg53Cys inhibits C3bBb formation to the same degree as WT FH, while p.Trp134Arg and p.Arg175Pro shows significant impairment in their ability to prevent C3 convertase formation. Results were normalized to CA and presented as relative protein expression. Box plots show median, 1^st^ and 3^rd^ quartile ranges, and the individual data points of three independent experiments. Significance calculated for WT FH. **(D)** Western blot analysis of CA formed alone, then decayed in buffer, or with WT FH or recombinant WT FH. There is no difference between the two FH conditions. **(E)** Western blot analysis of CA formed alone, then decayed in buffer, or with p.Arg53Cys or WT FH. p.Arg53Cys almost reached significance at 1 min as compared to WT FH *(p=*0.055*).* **(F)** Western blot analysis of CA formed alone, then decayed in buffer, or with p.Trp134Arg or WT FH. p.Trp134Arg (*t_1/2 =_* 2*_ min_*, *t_1/2 =_* 2.5*_ min_*) shows very poor DAA, providing only 20% decrease in C3bBb half-life as compared to CA. **(G)** Western blot analysis of CA formed alone, then decayed in buffer, or with p.Arg175Pro or WT FH. p.Arg175Pro (*t_1/2_* ~1.5 min, CA *t_1/2 =_* 3.1*_ min_*) decreases DAA as compared to WT FH. For all decay experiments, results were normalized to CA at 0 min and presented as relative protein expression. Ponceau S Staining was used as a measure of total protein load. Mean ± SD of three independent experiments. Statistical significance shown for comparisons between WT FH/recombinant FH at the same timepoint. * P ≤ 0.05, ** P ≤ 0.01, *** P ≤ 0.001, **** P ≤ 0.0001.

We performed functional studies on three reported pathogenic variants in *CFH* (**Figure 6A**). The C3 convertase formation assay was performed to assess the ability of recombinant WT FH (FH his) and FH mutants to prevent C3 convertase formation. Non-recombinant WT FH and FH his prevented C3 convertase formation equally well (**Figure 6B**). p.Arg53Cys also prevented C3bBb formation. In contrast, p.Trp134Arg and p.Arg175Pro showed diminished ability to inhibit C3 convertase formation as compared to WT FH (**Figure 6C**).

Next, we tested the decay accelerating activity of FH his and FH mutants. FH his was as effective as WT FH in accelerating C3 convertase decay (**Figure 6D**). C3 convertase decay rate in presence of p.Arg53Cys (CA *t_1/2 =_* 3.1*_ min_*) was not significantly different from WT FH (*P*=0.055) at 1 min (**Figure 6E**). The presence of either p.Trp134Arg (**Figure 6F**) or p.Arg175Pro (*t_1/2_* ~1.5 min, CA *t_1/2 =_* 3.1*_ min_*) (**Figure 6G**) exhibited a significantly slower DAA at 1 min as compared to WT FH. Notably, p.Trp134Arg mutant showed particularly poor DAA; its *t_1/2 =_* 2*_ min_* compared to untreated CA *t_1/2 =_* 2.5*_ min_*.

### Assessment of acquired drivers of C3G

#### IgG derived from C3Nef-positive C3G patients stabilized C3 convertase, some promoted formation

**Figure 7:**
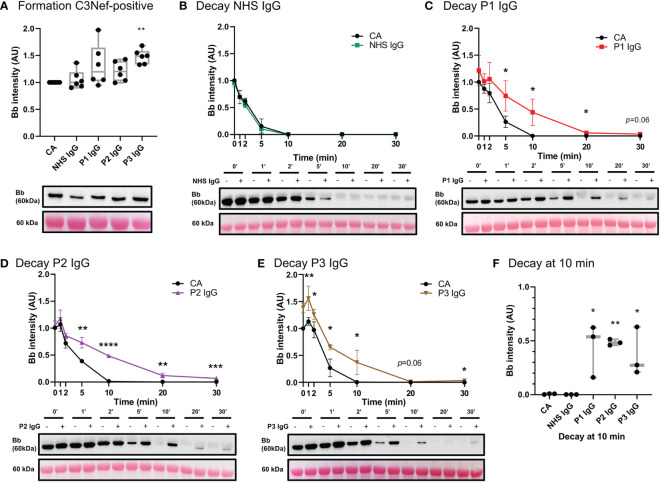
IgG from C3Nef+ C3G patients stabilize C3 convertase and decrease its decay rate. **(A)** Western blot analysis of CA formed alone, or in presence of NHS, P1, P2 or P3 IgG. P3 IgG significantly increases C3bBb formation. Results were normalized to CA and presented as relative protein expression. Box plots show median, 1^st^ and 3^rd^ quartile ranges, and the individual data points of six independent experiments. Significance calculated for NHS IgG. **(B)** Western blot analysis showing C3 convertase formed alone **(**CA) or in presence of NHS IgG, then decayed for up to 30 min. NHS IgG do not stabilize C3 convertase (NHS IgG t_1/2 =_ 3_ min_, CA t_1/2 =_ 3.36_ min_). **(C–E)** Western blot analyses showing C3 convertase formed alone (CA) or in presence of IgG derived from C3Nef+ patients, then decayed for up to 30 min. **(C)** P1 IgG stabilizes C3bBb 2-fold (P1 t_1/2 =_ 8.9_ min_, CA t_1/2 =_ 4.3min). **(D)** P2 IgG stabilizes C3bBb 1.9-fold (P2 t_1/2 =_ 9.4_ min_, CA t_1/2 =_ 4.9_ min_). **(E)** P3 IgG stabilizes C3bBb 1.7-fold (P3 t_1/2 =_ 8.7_ min_ CA t_1/2 =_ 5_ min_). Mean ± SD of three independent experiments. Statistical significance shown for comparisons between CA/Patient IgG at the same timepoint. **(F)** Composite box plots showing C3 convertase stabilization by IgG from C3Nef+ C3G patients after 10 min of decay in buffer. For all decay experiments, results were normalized to CA at 0 min and presented as relative protein expression. Ponceau S Staining was used as a measure of total protein load. Box plots show median, and the individual data points of three independent experiments. Significance calculated for NHS IgG at 10 min. * P ≤ 0.05, ** P ≤ 0.01, *** P ≤ 0.001, **** P ≤ 0.0001.

Antibodies against AP C3 convertase called C3 Nephritic Factors (C3Nefs) are a common driver of disease (2, 40). Here, we used C3 convertase decay and formation assays to assess the stabilization capacity of IgG derived from C3Nef-positive C3G patients (**Figure 7**) (**Table 1**). Addition of normal human serum IgG (NHS IgG) in C3CDA did not affect the decay rate of convertase (**Figure 7B**). Stabilization of C3 convertase was observed most prominently with P1 IgG (P1 t_1/2 =_ 8.9_ min_, CA t_1/2 =_ 4.3min) (**Figure 7C**) and P2 IgG (P2 t_1/2 =_ 9.4_ min_, CA t_1/2 =_ 4.9_ min_) (**Figure 7D**), which showed 2- and 1.9-fold increases in half-life, respectively. Addition of P3 IgG (P3 t_1/2 =_ 8.7_ min_ CA t_1/2 =_ 5_ min_) allowed for 1.7-fold increase in convertase half-life (**Figure 7E**). All C3Nef-positive patient IgG promoted C3 convertase stabilization past 10 min, as compared to NHS IgG (**Figure 7F**). Interestingly, P3 IgG consistently formed more C3 convertase in the C3CFA than did P1 or P2 IgG (**Figure 7A**).

**Figure 8:**
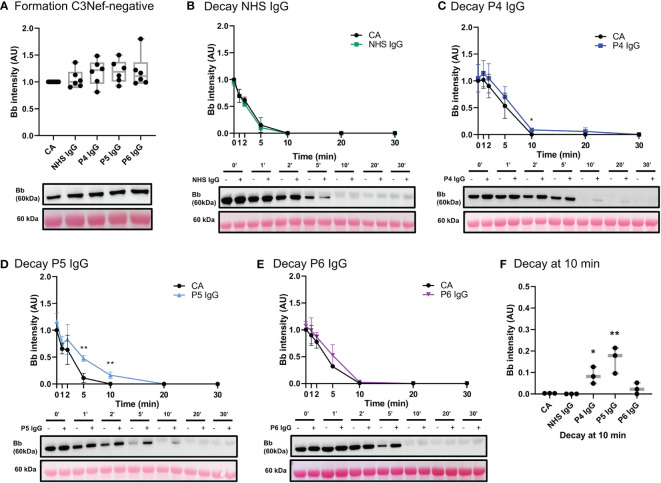
IgG from C3Nef- C3G patients do not promote C3 convertase formation but can increase its stability. **(A)** Western blot analysis of CA formed alone, or in presence of NHS, P4, P5 or P6 IgG. IgG from C3Nef-negative C3G patients does not affect C3bBb formation. Results were normalized to CA and presented as relative protein expression. Box plots show median, 1^st^ and 3^rd^ quartile ranges, and the individual data points of six independent experiments. Significance calculated for NHS IgG. **(B)** Western blot analysis showing C3 convertase formed alone (CA) or in presence of NHS IgG, then decayed for up to 30 min. NHS IgG do not stabilize C3 convertase (NHS IgG t_1/2 =_ 3_ min_, CA t_1/2 =_ 3.36_ min_). **(C–E)** Western blot analyses shows C3 convertase formed alone **(**CA) or in presence of IgG derived from C3Nef- patients, then decayed for up to 30 min. **(C)** P4 IgG increases C3bBb half-life 1.3-fold (P4 t_1/2 =_ 7_ min_, CA t_1/2 =_ 5.5_ min_). **(D)** P5 IgG increases C3bBb half-life 1.7-fold (P5 t_1/2 =_ 5.5_ min_, CA t_1/2 =_ 3.3_ min_). **(E)** P6 IgG increases C3bBb half-life 1.2-fold (P6 t_1/2 =_ 5.6_ min_ CA t_1/2 =_ 4.6_ min_). Mean ± SD of three independent experiments. Statistical significance shown for comparisons between CA/Patient IgG at the same timepoint. **(F)** Composite box plots showing C3 convertase stabilization by IgG from C3Nef-negative patients (P4, P5) after 10 min of decay in buffer. For all decay experiments, results were normalized to CA at 0 min and presented as relative protein expression. Ponceau S Staining was used as a measure of total protein load. Box plots show median, and the individual data points of three independent experiments. Significance calculated for NHS IgG at 10 min. * P ≤ 0.05, ** P ≤ 0.01, *** P ≤ 0.001, **** P ≤ 0.0001.

Three C3Nef-negative patient IgG (**Table 1**) showed increased stabilization of C3bBb in decay assays: 1.3-fold increase in C3bBb half-life in the presence of P4 IgG (P4 t_1/2 =_ 7_ min_, CA t_1/2 =_ 5.5_ min_), 1.7-fold with P5 IgG (P5 t_1/2 =_ 5.5_ min_, CA t_1/2 =_ 3.3_ min_) and 1.2-fold with P6 IgG (P6 t_1/2 =_ 5.6_ min_ CA t_1/2 =_ 4.6_ min_) (**Figure 8C–E**). While P6 IgG and P7 IgG allowed for some convertase stabilization at 10 min post formation (**Figure 8F**), it is evident that little to no C3bBb complexes were present on the ECM surface past that timepoint. The convertase formation assay showed no significant change in any C3Nef-negative patient IgG (**Figure 8A**).

## Discussion

In this body of work, we present an *in vitro* model of C3G that utilizes human ECM (MaxGel), purified human complement proteins, and patient-derived antibodies to improve our understanding of C3G pathogenesis and promote patient-specific diagnostics. The first step in the development and validation of this model was to ensure that any change in C3bBb observed was due to the proteins added and not potential contamination of the ECM. We show that MaxGel does not permit C3 convertase formation unless all three essential components of the convertase are added, and that fluid-phase complement regulators successfully inhibit (FH) or promote (FP) convertase formation (**Figure 3A**). These outcomes are consistent with previous studies (41–47) and confirm the expected negative and positive regulation of C3bBb on ECM surface by FH and properdin, respectively. We also show that when formed in presence of properdin, the decay of C3bBb is >2 times slower (**Supplemental Table 1**) as compared to untreated C3 convertase (**Figure 3B**). These results align with the findings of a classical 1975 study where C3 convertase assembled with FP on sheep erythrocyte surface was stabilized 1.5- to 10-fold (34). It is important to note that different surfaces used as a base for C3 convertase reconstitution have been shown to result in different C3bBb half-lives (plastic = ~90 sec (48), sheep erythrocytes ~4 min (34), MaxGel ~ 3.8 min), highlighting the importance of studying complement activation on a surface that is relevant to disease pathogenesis. Next, we also showed the expected dose-response of the addition of FH and/or FP on C3bBb regulation, as confirmed by their concentration gradients and a combined assessment of FH : FP ratios (**Figure 4**). The results of patient-specific FH : FP assessments aligned with the gradient curve established in lanes 4-9 (**Figure 4C**), suggesting that the FH : FP ratio alone is not necessarily a predictor of pathogenicity.

To assess the genetic drivers of C3G, the impact of three variants each in *CFB* and *CFH* on C3 convertase formation and decay was characterized. With respect to *CFB*, we largely recapitulated the described phenotypes (**Figure 5**). p.Arg138Trp and p.Asp279Gly caused loss-of-function and gain-of-function effects, respectively (38, 49). With p.Phe286Leu, we observed very few active C3bBb complexes; uncleaved C3bB (50, 51) complexes were present instead, even when formed and decayed with FH. These data suggest that under physiological concentrations of FB and FD, p.Phe286Leu FB may have a high affinity for C3b while not being successfully cleaved by FD. A similar phenotype of p.Phe286Leu was shown in a 2007 study, where this mutant’s high affinity for C3b and decreased cleavage by FD was hypothesized to result in generation of abundant, rapidly cycling C3 convertase if supplied with unlimited FB (39). Considering both sets of data, we instead hypothesize that under normal physiological concentrations, the impaired cleavage of FB p.Phe286Leu by FD compensates for its increased affinity towards C3b. In light of a recent finding describing FD-independent AP activation (52), we also hypothesize that the incorporation of highly decay-resistant FB p.Phe286Leu into C3(H_2_O)B may lead to increased C3 cleavage over time, thus propagating the complement amplification process.

C3 convertase formation and decay assays were used to assess three well-characterized pathogenic variants (p.Arg53Cys, p.Trp134Arg, p.Arg175Pro) in short consensus repeats 1-3 of *CFH*. Their respective inhibitory functions recapitulated the results from the previous studies (**Figure 6**) (23, 24).

We next assessed the acquired drivers of C3G. IgG from six C3G patients was assessed for C3bBb stabilization capacity (**Table 1**), and a spectrum of outcomes was found. While showing no effect on convertase formation rate, some IgG derived from C3Nef-negative patients had a mild stabilizing effect on C3bBb up to 10 min post formation. This finding suggests that some IgG derived from C3Nef-negative patients may stabilize C3bBb for a short period of time, likely contributing to the overall disease pathogenesis when other disease factors are present. Assessments of C3Nef-positive patient-derived IgG resulted in expected increases in C3 convertase half-life, consistent with described C3Nef functions (3, 18, 20). Importantly, unlike any other IgG tested, P3 IgG increased C3bBb formation while providing weak stabilization (**Table 1**), suggesting a novel method of C3G pathogenesis whereby C3Nefs promote increased formation of C3bBb.

There are two main limitations to the proposed model. First, while MaxGel is a human basement membrane extract, we cannot ensure that it recapitulates the exact composition of the ECM found in human kidneys. Differences in glycosaminoglycan composition may need to be considered when evaluating convertase regulation by FH and FP on the surface of these ECM. Second, His-tags on the recombinant proteins may change complement dynamics and pathway activity, as demonstrated by the His-tagged WT FB (**Figure 5B**, lanes 3-4; **5C**). Decreased C3bBb formation and faster convertase decay with recombinant WT FB indicate the need to control for the effects of His-tags on these processes and adjust the data interpretation accordingly.

In summary, we have developed a new model to test complement activity and regulation on an ECM surface. This model recapitulates normal complement activity and when used to test both genetic and acquired drivers of C3G, provides valuable insights into how complement activity can be altered in this microenvironment. Its further applications to complement-mediated glomerular diseases may facilitate patient-specific insights into disease pathogenesis.

## Data availability statement

The original contributions presented in the study are included in the article/**Supplementary Material**. Further inquiries can be directed to the corresponding author.

## Ethics statement

The studies involving human participants were reviewed and approved by Institutional Review Board of the University of Iowa. The patients/participants provided their written informed consent to participate in this study.

## Author contributions

RS and YZ conceived the study. SP and XX designed the protocol. SP troubleshot the protocol, designed and carried out the experiments, analyzed the data, made the figures and drafted the manuscript. NM and RG quantified C3Nefs. RS, YZ, CN, NM and SP revised the manuscript. All authors contributed to the article and approved the submitted version.
